# Robust Hand Motion Tracking through Data Fusion of 5DT Data Glove and Nimble VR Kinect Camera Measurements

**DOI:** 10.3390/s151229868

**Published:** 2015-12-15

**Authors:** Ewout A. Arkenbout, Joost C. F. de Winter, Paul Breedveld

**Affiliations:** Department of Biomechanical Engineering, Faculty of Mechanical, Maritime and Materials Engineering, Delft University of Technology, Mekelweg 2, 2628 CD Delft, The Netherlands; J.C.F.deWinter@tudelft.nl (J.C.F.W.); P.Breedveld@tudelft.nl (P.B.)

**Keywords:** Human-computer interaction, Kalman filter, data fusion, gestures, finger joint angle measurements, sensor redundancy

## Abstract

Vision based interfaces for human computer interaction have gained increasing attention over the past decade. This study presents a data fusion approach of the Nimble VR vision based system, using the Kinect camera, with the contact based 5DT Data Glove. Data fusion was achieved through a Kalman filter. The Nimble VR and filter output were compared using measurements performed on (1) a wooden hand model placed in various static postures and orientations; and (2) three differently sized human hands during active finger flexions. Precision and accuracy of joint angle estimates as a function of hand posture and orientation were determined. Moreover, in light of possible self-occlusions of the fingers in the Kinect camera images, data completeness was assessed. Results showed that the integration of the Data Glove through the Kalman filter provided for the proximal interphalangeal (PIP) joints of the fingers a substantial improvement of 79% in precision, from 2.2 deg to 0.9 deg. Moreover, a moderate improvement of 31% in accuracy (being the mean angular deviation from the true joint angle) was established, from 24 deg to 17 deg. The metacarpophalangeal (MCP) joint was relatively unaffected by the Kalman filter. Moreover, the Data Glove increased data completeness, thus providing a substantial advantage over the sole use of the Nimble VR system.

## 1. Introduction

The use of hand gestures as a control input in Human-Computer Interaction (HCI) is an ongoing topic of research [1,2,3,4]. In human-to-human interaction, hand movements are a means of non-verbal communication, and can take the form of either simple actions (such as pointing to an object) or more complex ones (such as when expressing feelings). Therefore, it stands to reason that using the hands can be an intuitive method for the communication with computers. The hands can be considered an input device with more than 20 degrees of freedom (DOF) [3,5], as such it should be possible to use the hands as high DOF control devices in a wide range of applications.

Two major types of technology for HCI can be distinguished, namely contact based and vision based devices. Contact based devices rely on physical interaction with the user. Vision based devices, on the other hand, analyze one or more video streams for determining hand motions. Examples of contact based devices are mobile touch screens (e.g., for monitoring, communication and guidance on an industrial shop floor [6]) and data gloves (e.g., for tracking of the hands in computer animations [7,8]). Most vision based devices fall into the categories of interactive displays/table tops/whiteboards, robot motion control, and sign language [2]. For example in the automotive domain, the use of hand gestures can be a valuable asset for the control of interfaces that would otherwise require physical interaction with the driver [9]. In the medical domain, vision based devices have been researched as a non-contact substitute for the mouse and keyboard, allowing the surgeon to interact with computers in a sterile environment. For example, Graetzel *et al.* [10] enabled the surgeon to perform standard mouse functions through hand gestures, and Rosa and Elizondo [11] used the recently introduced Leap Motion (Leap Motion Inc., San Francisco, CA, USA) [12,13,14] to provide intra-operative touchless control of surgical images.

A large number of *contact based* data gloves have been developed over the last 35 years [15], whereas vision based tracking of the hands has been in development for about two decades [3,16,17]. The application of vision based devices is of interest, as cameras are becoming more and more prevalent, featuring continually increasingly sampling rates and an exponentially growing number of pixels [18,19]. However, pressing challenges in vision based hand gesture recognition are to cope with a large variety of gestures, hand appearances, silhouette scales (spatial resolution), as well as visual occlusions [1,3]. In comparison, contact based devices are easy to implement, but require calibration because the measurement is relative rather than absolute with respect to the earth.

Gloves and camera systems each have their limitations, but may complement each other. Sensor fusion of a vision based with a contact based device has several advantages, in particular that the contact based device can fill in the data gap that occurs with vision based systems during camera occlusions, and that the vision based device provides an absolute measurement of hand state. Moreover, the fusion of data can result in a higher precision of pose estimates through redundancy gain.

Previous research has integrated two vision based systems for the purpose of high fidelity hand motion data acquisition [20]. Furthermore, various studies have integrated vision and contact based systems with the aim of aiding in the tracking of the location of a grasped object within a hand [21,22,23,24] or for improving the recognition of sign language and hand gestures [25,26,27]. These multi-sensor techniques supplement each other, where the separate sensors measure different aspects of the motions of the arm and hands, after which their combined data is used for higher-level feature extraction for gesture recognition [28]. However, using sensor redundancy and fusion with the primary purpose of increasing precision and robustness of a vision based hand posture approximation is rarely performed.

Because of the inherent issue of visual occlusions associated with cameras, updating the hand posture approximation with local sensors may often be necessary. A recommendation in this regard is to use as few and as minimally obtrusive sensors as possible, thereby not influencing natural hand and finger motions. Accordingly, this research presents a simple method for fusing contact based with vision based hand tracking systems, where the focus is placed on using a camera tracking system that is readily available, and a data glove that uses a small number of sensors.

### 1.1. HCI in Laparoscopic Training

A field where hand motions and postures as HCI input may be promising is virtual laparoscopic training. Various medical trainers exist for laparoscopic skills training and assessment, ranging from physical box trainers to high fidelity virtual reality (VR) trainers [29], both of which are effective training devices [30,31,32]. Contemporary virtual simulators need a physical interface, both for purposes of congruence with the actual operating room scenario as well as for reliable tracking of the hand motions. These VR trainers usually aim to simulate the minimally invasive surgical scenario as realistically as possible. Surgeons in training may benefit from practicing with such realistic systems, but due to the considerable cost gap between VR simulators and physical box trainers, the use of VR simulators is currently limited to a relatively small number of training centers [33]. As such, it may be beneficial to have a cheaper VR alternative.

Comparing the medical field to training methods in aviation, one can see that it is standard practice to train pilots in simulators of increasing complexity, where basic tasks are trained in lower fidelity and part-task simulators. For example, Integrated Procedures Trainers (IPTs) allow for the learning of flow patterns, systems, procedures, and checklists [34]. In the same way, one could train surgeons, starting out with a virtual trainer that simulates basic laparoscopic tasks to train hand–eye coordination skills, for example in a scenario where instrument movements are inverted with respect to the hand movements (*i.e*., the “fulcrum effect” associated with the entry incision). Training of these basic laparoscopic skills is to a certain extent possible without the need for a physical interface, making visual based tracking devices potentially useful for the early training of surgeons. Depending on the skills that the surgeon aims to learn, a certain level of precision and accuracy of the hand state estimate is required. However, these requirements may be relaxed when learning basic spatial abilities, for example when learning to control an instrument with inverted movement [35,36] or when learning to use an angled laparoscope [37].

Using a vision based device for virtual laparoscopic training may furthermore be interesting in light of the recent surge in low cost consumer market VR headsets (*i.e.*, Oculus Rift [38], Sony PlayStation VR [39], HTC Vive [40], and Samsung Gear VR [41]), which are devices that could enhance the fidelity of medical VR simulators. Such high fidelity VR simulations may confer effective skills transfer to the *in vivo* surgical situation, whereas less expensive VR trainers may lead to effective skill generalization [42,43,44,45]. Unfortunately, as previously mentioned, the limited accuracy and precision of current vision based devices for tracking of the hand movements, as well as their inherent issue of visual occlusions, makes them not yet suitable for surgical applications. Both issues may be solved through the integration of a contact based device.

### 1.2. Nimble VR

A relatively new vision based system is the Nimble VR (Nimble VR Inc., San Francisco, CA, USA), previously named 3Gear Systems. It currently relies on the Microsoft Kinect^TM^ sensor (Microsoft Corporation, Redmond, WA, USA) and obtains the hand pose estimates through queries of a precomputed database that relates the detected hand silhouettes to their 3D configurations [46]. The Microsoft Kinect has a QVGA (320 × 240 px) depth camera and a VGA (640 × 480 px) video camera, both of which can produce image streams up to 30 frames per second [47]. Moreover, the Kinect has a horizontal and vertical field of view of 57 and 43 degrees, respectively, with a depth sensor range of 1.2 m to 3.5 m.

Previous research into the Nimble VR system has shown the measurement errors of the position of the hand to depend on the distance from the camera [48] and the variance of the measurement data to depend on the orientation of the hand [49]. Kim *et al.* [48] evaluated the Nimble VR (v.0.9.21) and concluded that it did not provide results of high enough accuracy and robustness over the working range that is required for a medical robotics master. Continued development, however, as well as the addition of data filtering, smoothing, and downscaling of motions, can improve the performance of this vision based system [48,50,51].

The goal of the present research is to implement a Kalman filter algorithm to fuse measurement data of the vision based Nimble VR system with a contact based measurement system, for research into the use of hand and finger motions in medical VR simulators. As previously mentioned, a requirement for the contact based device is that it should be minimally obtrusive to the surgeon, because physical sensors may impede the naturalness of motion and therefore influence surgical VR skills training. We selected the 5DT Data glove (5th Dimension Technologies, Irvine, CA, USA) [52], providing five basic full finger flexion sensors. Although this data fusion approach negates the contact-free control advantage that characterizes vision based systems, it allows for improved pose estimates at visual occlusions and a higher update frequency due to a higher sampling rate of the Data Glove (200 Hz) as compared with the Nimble VR (currently running at 15 Hz). This study presents the implementation of the filter as well as its validation. The validation was performed through measurements of the finger joint angles of a wooden hand model in various poses and orientations. The pose estimates from the 5DT Data Glove, Nimble VR, and the filter were assessed with respect to the actual finger joint angles of the hand model. Additionally, dynamic finger flexion angles, measured on three differently sized hands, were performed, and the data with and without implementation of the filter were compared.

## 2. Kalman Filter Procedures and Parameter Settings

The Kalman filter is a computationally efficient recursive solution of the least-squares method, supporting estimates of the past, present, and future states of a modeled system [53].

In this research, we used the Kalman filter to combine Nimble VR measurements with local sensor data obtained from the 5DT Data Glove 5 Ultra [15,52]. The Data Glove allows for the measurement of overall flexion of each finger by means of fiber-optics-based bending sensors. Although the Data Glove does not distinguish between the individual finger joints, it does have the advantage of being independent of hand orientation and hand position. Moreover, because the Data Glove uses only five simple sensors, it does not significantly impede hand movements. Fusing the local sensor data with the obtained global camera data has the expected advantage of increasing data completeness during hand occlusion and during hand orientations at which the camera-based tracking system is unable to provide an accurate estimation.

The Kalman filter has already been described extensively in the literature [54,55]. The basic equations are as follows:

Measurement update equations:

(1) $$K_{k}=P_{k}^{-}H_{k}^{T}{\left(H_{k}P_{k}^{-}H_{k}^{T}+R_{k}\right)}^{-1}$$

(2) $${\hat{x}}_{k}={\hat{x}}_{k}^{-}+K\left(z_{k}-H_{k}{\hat{x}}_{k}^{-}\right)$$

(3) $$P_{k}=\left(I-K_{k}H_{k}\right)P_{k}^{-}$$

Time update equations:

(4) $$x_{k+1}^{-}=A_{k}{\hat{x}}_{k}+Bu_{k}$$

(5) $$P_{k+1}^{-}=A_{k}P_{k}A_{k}^{T}+Q_{k}$$

where $K_{k}$ is the Kalman gain, $P_{k}$ the estimate error covariance matrix, $H_{k}$ the matrix describing how the measurement equation relates to the actual measurement $z_{k}$, $A_{k}$ contains the model functions describing the relation between the state at time step *k* and the state at step *k + 1*, and B the matrix relating control input $u_{k}$ to the state x. In our case, the state vector x contains the metacarpophalangeal (MCP) and proximal interphalangeal (PIP) joint angles and angular velocities for each finger. The actual measurements $z_{k}$ are limited to the MCP and PIP joint angles individually as obtained through the Nimble VR software and the sum of the two as given by the Data Glove. Vectors x and $z_{k}$ and matrices $H_{k}$, $A_{k}$ and B are given as follows:

(6) $$\begin{array}{l} x=\left[\begin{array}{c} \varphi_{MCP}\\ \varphi_{PIP}\\ {\dot{\varphi }}_{MCP}\\ {\dot{\varphi }}_{PIP} \end{array}\right]z_{k}=\left[\begin{array}{c} \varphi_{NVR_{MCP}}\\ \varphi_{NVR_{PIP}}\\ \varphi_{DG_{MCP+PIP}} \end{array}\right]\begin{array}{c} \}\text{Nimble VR (NVR)}\\ \}\text{Data Glove (DG) } \end{array}H_{k}=\left[\begin{array}{cccc} 1 & 0 & 0 & 0\\ 0 & 1 & 0 & 0\\ w_{DG_{MCP}} & w_{DG_{PIP}} & 0 & 0 \end{array}\right]\\ \begin{array}{ccc} A_{k}=\left[\begin{array}{cccc} 1 & 0 & dt & 0\\ 0 & 1 & 0 & dt\\ 0 & 0 & 1 & 0\\ 0 & 0 & 0 & 1 \end{array}\right] & B=0 & \left(\text{null matrix}\right) \end{array} \end{array}$$

Note that these matrices are valid for all fingers, with the exception that the thumb has an interphalangeal (IP) joint instead of a PIP joint. The carpometacarpal (CMC) joint of the thumb is not measured with the Data Glove, and therefore not present in this model. The distal interphalangeal (DIP) joint is not measured by either of the two systems, because these joints are linked in motion to the PIP joints and because one cannot easily control one’s own DIP joints. Hence, these joints were left outside the scope of this research. The B matrix is a null matrix because we do not provide a custom input u.

Matrix $H_{k}$ contains two weights $w_{DG_{MCP}}$ and $w_{DG_{\mathrm{PIP}}}$ which represent the degree to which the respective finger joints contribute to the Data Glove measurement signal. The measurement error covariance matrix $R_{k}$, the process noise covariance matrix $Q_{k}$ and the Data Glove weights $w_{DG_{MCP}}$ and $w_{DG_{\mathrm{PIP}}}$ were measured prior to operation of the filter, and are described next.

### Determining the Kalman Filter paRameters

Research has shown that the mean finger flexion obtained from Nimble VR (v0.9.34) measurements is dependent on the orientation of the hand [49]. The level of variance for each finger joint as a function of both hand orientation and the degree of finger flexion serves as input for the measurement error covariance matrix $R_{k}$:

(7) $$R_{k}\left(\alpha ,\beta ,\gamma ,z_{k}\right)=\left[\begin{array}{ccc} \sigma_{NVR_{MCP}}^{2}\left(\alpha ,\beta ,\gamma ,z_{k}\right) & 0 & 0\\ 0 & \sigma_{NVR_{PIP}}^{2}\left(\alpha ,\beta ,\gamma ,z_{k}\right) & 0\\ 0 & 0 & \sigma_{DG_{MCP+PIP}}^{2} \end{array}\right]$$

where $\sigma_{NVR_{MCP}}^{2}\left(\alpha ,\beta ,\gamma ,z_{k}\right)$ and $\sigma_{NVR_{PIP}}^{2}\left(\alpha ,\beta ,\gamma ,z_{k}\right)$ are the Nimble VR measured MCP and PIP joint angle variances as a function of pitch, roll, and yaw angles (α,β,γ, respectively) and Data Glove measurement $z_{k}$. $\sigma_{DG_{MCP+PIP}}^{2}$ is the data variance associated with the Data Glove, which is independent of hand orientation. The off-diagonal elements are the correlations between the various joints. Because a person can actuate their MCP and PIP joints independently of each other (to a certain degree), these elements were set to zero. The correlations between the different fingers were set to zero for the same reason. The variance terms used as input for the Kalman filter were measured as a function of hand orientation and finger flexion. The method by which this has been done and the accompanying results are given in Appendix A.

The noise covariance matrix $Q_{k}$ is typically used to represent the uncertainty in the process model [53]. We set this uncertainty to be equal to the squared angular deviation from the state estimation ${\hat{x}}_{k}$, as calculated with the peak rotational acceleration of the finger flexions. Because changes in finger flexion cannot be greater than the physical maximum during voluntary free finger movement, this approach provides us with a valid uncertainty range for where a finger can be at a point in time based on its previous location. The process noise covariance matrix $Q_{k}$ then becomes:

(8) Qk=[(14)φ¨MCP2⋅δt40(12)φ¨MCP2⋅δt300(14)φ¨PIP2⋅δt40(12)φ¨PIP2⋅δt3(12)φ¨MCP2⋅δt30φ¨MCP2⋅δt200(12)φ¨PIP2⋅δt30φ¨PIP2⋅δt2]

where ${\ddot{\varphi }}_{MCP}$ and ${\ddot{\varphi }}_{PIP}$ are the maximum joint angular accelerations. The values used as input for this matrix were measured experimentally and are provided in Appendix B.

Lastly, because the Data Glove measures the sum of MCP and PIP flexion, the following relation holds for the two weights $w_{DG_{MCP}}$ and $w_{DG_{\mathrm{PIP}}}$:

(9) $$\varphi_{DG_{MCP+PIP}}=w_{DG_{MCP}}\cdot \varphi_{MCP}+w_{DG_{PIP}}\cdot \varphi_{PIP}$$

Ideally, the weights have a value of 1.0 each, indicating proper measurement of the individual joint rotations. However due to shifting of the Data Glove sensors inside the glove with respect to the fingers, the measurement signals may be biased and vary per finger. Hence, these weights were measured using a medium sized hand prior to the Kalman filter operation. The measurement procedures and resulting weight values are provided in Appendix C.

## 3. Methods

### 3.1. Test Setup

**Figure 1:**
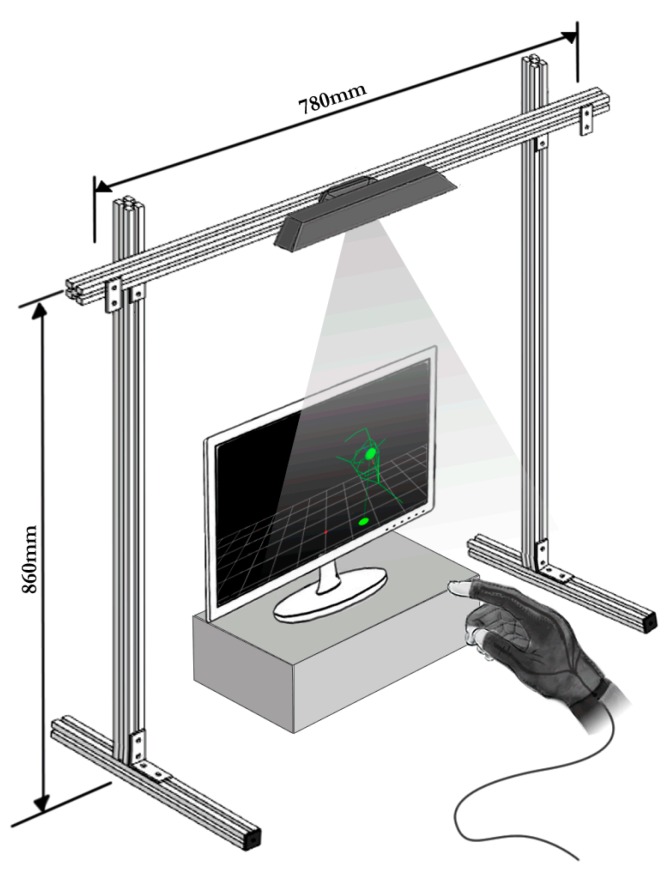
Schematic representation of the test setup.

A setup was created that implements the Nimble VR camera-based hand tracking software (v0.9.36). This setup made use of a Kinect camera mounted on a rig facing downwards onto a table top (Figure 1). Using the infrared depth information obtained from the Kinect camera, the software detected the hands, provided an estimation of the orientation and position of the hands and fingers, and approximated the hand’s skeletal model [56]. Default software settings were used. The 5DT Data Glove was added to this setup, and we wrote a C++ program that exports all measurements to MATLAB. In MATLAB, the Kalman filter function fused the Nimble VR and Data Glove measurements.

After implementing the predetermined filter parameters (*i.e.*, the measurement error covariance matrix $R_{k}$, the process noise covariance matrix $Q_{k}$, and the Data Glove weights $w_{DG_{MCP}}$ and $w_{DG_{\mathrm{PIP}}}$, see Appendix A, Appendix B and Appendix C), the Kalman filter output was compared with the Nimble VR measurements.

Two validation measurements were performed. The first measurements used a wooden model hand to assess the influence of hand orientation on the Nimble VR output, and to assess the degree to which the Kalman filter is able to improve the state estimates by fusing with the Data Glove output. The second measurements involved dynamic hand movements with three human hands of different sizes to assess the robustness of the Kalman filter output. This is important because predetermined Kalman parameters were used in combination with a single one-size-fits-all glove. Moreover, the dynamic measurements provide a measure of the time delay of the current setup.

### 3.2. Wooden Hand Model Measurements to Validate the Kalman Filter Operation

The wooden model hand, which is widely available for purchase, had a length of 21.35 cm, measured from wrist to tip of the middle finger and breadth of 8.3 cm (note that the same model was used for determining matrix $R_{k}$, see Appendix A). Using a real hand for these measurements was not possible, because a human cannot keep his hand in a constant position during time consuming measurements. Using a model hand offered good experimental control, and moreover enables the current study to be reproduced and the results to be compared to later iterations of the Nimble VR software, different software packages, or the use of alternative cameras.

The model, mounted on a tripod with a three-way pan/tilt head, was placed in five different postures, while wearing the glove in view of the Nimble VR system. As is standard practice, the Data Glove was calibrated to the full range of motion of the hand [52]. Flat hand, pure MCP flexion, pure PIP flexion, combined MCP and PIP flexion, and pinch grip postures were assessed, and the data from the Nimble VR system was compared with the Kalman filtered results. The orientation of the hand model was varied by placing it in varying pitch, roll and yaw angles (ranges: [−60, 30] deg, [−120, 60] deg, and [−60, 60] deg, respectively). These three angles were varied at 5 deg intervals, while keeping the other two angles constant at 0 deg. Five measurements were performed for each of the 5 postures and for each of the 3 ranges, where at each orientation angle 200 samples were collected (representing about 13 seconds of data at a mean frequency of 15 Hz). A measurement thus represents a full sweep through a chosen orientation range, and this entire sequence was repeated five times. The total number of measurements performed per posture for the pitch range for example was therefore 19,000 (= 200 samples * 19 angles * 5 repetitions). In total, 19 pitch angles, 25 yaw angles, and 38 roll angles were assessed. The roll measurements were performed in two separated sessions (ranges [−120, −30] and [−30, −60]), where the model hand was rotated 90 deg in between. As a result, one roll angle was measured twice (angle of −30 deg). The Data Glove was recalibrated at each change of hand posture to account for potential shifting of the sensors inside the glove, caused by the external forces applied to glove during the changing of hand postures. Note that during contactless measurements with human hands this recalibration is not needed as external forces potentially causing sensor shift should be absent. However, in practice the glove can easily be recalibrated in between measurement sessions if sensor drift is observed. The tripod was kept horizontally aligned with the test setup, with the hand rigidly attached to its pan/tilt head.

### 3.3. Human Hand Measurements to Validate the Kalman Filter Operation

Following the wooden hand model measurements, dynamic finger flexions were conducted on three different sized hands of healthy volunteers, ranging from small to large. Hand scale values, as automatically detected by the Nimble VR software, were 0.81, 0.84 and 0.87, respectively. The hand lengths, measured from wrist to tip of the middle finger, were 16.5 cm, 18.6 cm, and 19.6 cm, and breadth were 8.4, 9.2 cm, and 10.0 cm, respectively. In these tests, an additional Marker Tracking camera was used. This camera, capturing RGB data at 30 Hz with a resolution of 640 × 480 pixels, was aimed at the side of the hand. The positions of colored markers, attached to the joint locations of the index, pinky, and thumb fingers of the Data Glove, were extracted from the camera footage using RGB threshold and Mean Shift Cluster detection [57]. Calculating the joint angles from the marker locations provided a reference to which the Nimble VR and Kalman Filtered data could be compared. Marker Tracking analysis was not performed online, hence the tracking results are free from any time delay. More information on this Marker Tracking algorithm is provided in Appendix C. The joint angles were measured during active finger flexions with the Marker Tracking, Data Glove, and Nimble VR system. Each of the three participants performed five sets of ten repetitive full finger flexions (*i.e.*, from flat hand to closed fist and back) at a relaxed pace with the palm of the hand facing down. In between each set of ten flexions, the participant was asked to move his/her hand freely before going to the next set. A single glove calibration was performed prior to the measurements, calibrating the measurement range of the glove sensors to the movement range of the fingers of the participant.

### 3.4. Dependent Measures

The dependent measures are the precision, accuracy, completeness of the data, and time delay. Completeness was defined as the ability to provide (reliable) joint angle estimates of the joints of the fingers as a function of orientation, posture, and degree of visual self-occlusion of the hand. In the analyses, a distinction was made between the MCP and PIP joints of the fingers.

For the wooden hand model test, at each 5 deg step, the mean orientation of the hand was calculated over the 200 samples. The mean hand orientation angle per step was calculated by averaging over the 25 measurement sets performed (*i.e.*, 5 per posture and 5 different postures). The standard deviation (SD) was calculated as the mean of the 25 standard deviations.

Regarding the joint angle, at each 5 deg step of the hand orientation angle, the mean and standard deviation of the joint angle were calculated over the 200 samples. These values were then again averaged over the 5 measurement sessions performed for each of the 5 deg steps. This procedure was performed for each orientation range and each of the five postures.

Comparing the resulting mean joint angles to the actual joint angles provides a measure of the accuracy of the system. The standard deviation (and variance) around these mean joint angles are measures of precision. Additionally, comparing these performance measures of the Nimble VR system with the Kalman filtered data gives insight into the completeness of the data as a function of hand orientation and posture. At hand orientations where visual self-occlusion degrades the joint angle approximations, we expected for the Kalman filtered data lower standard deviations as well as more accurate joint angle approximations compared to the Nimble VR.

Independent two-sample *t* tests were performed to assess whether the difference in mean calculated joint angles between Nimble VR and the filter output were statistically significantly different from each other. The compared vectors (being of equal lengths) were each composed of the mean joint angles, calculated at each of the five measurements. A *t* test was performed between these vectors for each posture at every orientation angle, totaling 984 tests (index: 82 orientation angles * 5 postures * 2 joints; thumb: 82 angles * 1 posture * 2 joints). The accompanying degrees of freedom in each of the *t* tests was 8 (*i.e.*, *df* = 2*n* − 2, with *n* = 5). A *p* value smaller than 0.01 was deemed statistically significant. We selected a conservative significance level in order to reduce the probability of false positives.

In order to assess the overall benefit of the Kalman filter with respect to the Nimble VR system, we calculated for each orientation range the mean of the mean and the mean of the standard deviations taken over the entire range (the pitch, yaw and roll sample sizes were 19, 25 and 38, respectively). This represents the accuracy and precision respectively of the measurements for a specific pose and orientation range.

For the human hand test, the time delay of both the Nimble VR system and the Kalman filter was compared to the Marker Tracking measurements, which were not performed online, but were obtained through video post analysis. Hence, the Marker Tracking results are free from time delay. The root-mean-square error and the Pearson’s correlation coefficient of the Nimble VR and Kalman filter output with respect to the Marker Tracking measurements were calculated. Lastly, the maximum angular under- or overestimation of the measurement systems, occurring at full finger flexion, were extracted.

## 4. Results

**Figure 2:**
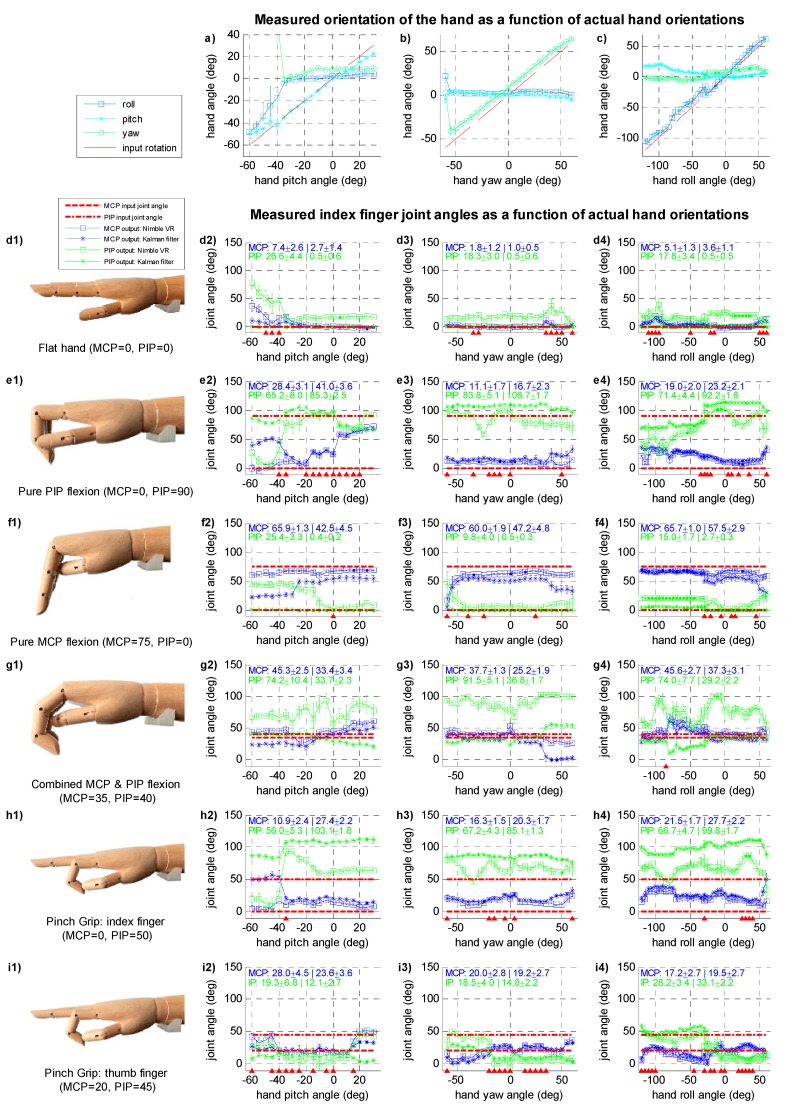
(**a–c**) Three plots providing measured orientation of the wooden hand model for varying actual hand orientations (*i.e.*, pitch, yaw, and roll), indicated by red dashed unity lines; (**d1–i1**) Photos of the measured hand orientations; (**d2–i4**) Plots showing the measured metacarpophalangeal joint (MCP, blue) and proximal interphalangeal (PIP, green) joint angles, as determined with the Nimble VR system (square markers, □) and after fusion of the Data Glove data through the application of the Kalman filter (asterisk markers, *). Data is presented with error bars ranging from mean – 1·SD to mean + 1·SD. The actual angles at which the fingers were placed are indicated with the red dotted and dash-dotted lines, and are illustrated in the photos provided on the left. All plots show data collected on the index finger, unless otherwise specified below the photo on the left. Indicated in the top left of every graph are the mean and standard deviation (format: mean ± SD | mean ± SD) calculated over the entire hand orientation range before (left) and after implementation of the Kalman filter (right), for both joints. Note that these mean and standard deviations are calculated as the mean of the mean, and the mean of the standard deviations, calculated per 5 deg step. The triangle markers on the horizontal axes indicate whether the difference in mean PIP joint angle between Nimble NR and the Kalman filter is not statistically significant (note: MCP joint is not visualised in this way).

The results for the posture measurements are shown in Figure 2 as a series of plots. The top three plots indicate the measured orientation of the hand model as a function of the input hand orientations, that is, across the pitch, yaw, and roll ranges. The remainder of the plots in Figure 2 show, for each of the hand postures, the index MCP joint angles (in blue) and PIP joint angles (in green) as a function of the actual hand orientation.

A distinction is made between the joint angles determined with the Nimble VR software only (square markers) and joint angles determined with the Kalman filter (asterisk markers). The actual joint angles of the wooden hand model are represented by horizontal lines (MCP: dashed line; PIP: dash-dotted line). Measurements lying closer to these lines are by definition more accurate. In each plot, at the top left corner, two means and two standard deviations are shown per joint. The first mean and standard deviation are that of the Nimble VR joint angle measurements taken over the entire range, and the second mean and standard deviation are that of the Kalman filtered data. A mean that lies closer to the actual joint angle indicates an overall improvement in joint angle approximation accuracy, and a lower standard deviation indicates an improvement in precision. Lastly, a solid triangle marker on the horizontal axes was used to indicate that the difference between the mean joint angle of the PIP joint obtained with Nimble VR and the Kalman filter is not statistically significant. This same display-method was not used for the MCP joint, because for this joint the measured angles were the same in approximately 50% of the cases, thereby cluttering the graphs if we were to display this.

### 4.1. Wooden Hand Model Orientation Measurements

The measured hand orientation angles are shown as a function of actual hand angle in Figure 2a–c. There are several orientation ranges of the hand at which the measurements of the hand angles are imprecise. For the pitch orientation (Figure 2a), all measured angles below −35 deg show very large standard deviations, that is, when the hand was pitched far downwards. The yaw angles (Figure 2b) show high precision for the range [−50, 50] deg. At angles below −50 deg, when the thumb was angled away from the screen, the measurements show large standard deviations, whereas above 50 deg the standard deviation increases slightly. Lastly, for roll angles (Figure 2c) in the range [−110, −60] deg, the measured angles have slightly larger standard deviations, which is because of visual occlusion of the fingers. At −90 deg, the hand is vertically aligned with the thumb on top. In this condition, the observed surface area of the hand is small, and only the thumb and index fingers can be distinguished by the Nimble VR software. As a result, in this range the orientation measurement becomes somewhat less reliable.

For the yaw measurements, a constant mean difference of about 9 deg is observed between the measured and actual yaw angle. Moreover, for the roll measurements a misalignment is seen at −30°, which is on account of the roll orientation having been measured in two separate sessions. A slight drift from actual the actual roll angle can be seen in the first range, where at −30 deg the measurement was stopped, the hand rotated 90 deg and reoriented, and the measurements (as well as the software) reinitialized. The re-measured roll angle of the hand is then free from drift and closer to the actual angle.

### 4.2. Wooden Hand Model Finger Joint Measurements—Index and Thumb Fingers

At hand orientations yielding a low precision (SD > 5 deg, see Figure 2, graphs a to c), a similar effect on precision can be seen for most of the finger joint angle estimates of the Nimble VR. The consequence of imprecise hand orientation measurements is either a decrease in finger joint angle estimation precision (*i.e.*, SD > 10 deg) or an unrealistically high precision (*i.e.*, SD < 1 deg) combined with a poor accuracy (> 30 deg shift from the true angle). This high precision is the result of visual self-occlusion of the finger, and the Nimble VR software accordingly making an estimation of the joint angles based on the estimated posture. This can for example clearly be seen in Figure 2, graph f2, at angles −60 to −15 deg, where for the PIP joint a 50 deg difference is observed between measured and true angle (thus having a low accuracy), while the observed precision is around 1 deg. Due to the orientation independent standard deviation of the Data Glove, the Kalman filter output has a low standard deviation, even when the standard deviation of the Nimble VR data is high. Furthermore, because the Data Glove output is independent of the hand orientation, the Glove contributes to improved accuracy of the Kalman filter output over all the hand orientation ranges, in particular for the PIP joint. In order to assess both the accuracy and the precision before and after implementation of the filter, Figure 2, subfigures d2-f4, should be referred to, as accuracy and precision are dependent on hand pose and assessed orientation range. The accuracy for a given joint, hand pose, or orientation range is equal to the mean joint angle (provided in the top left of every graph) minus the true joint angle. The precision is given by the standard deviations provided in the top left of every plot.

Looking at the index MCP joint, the two mean joint angles lie close together, and although an improvement in precision can be seen, only 49% of the time a significant difference between the measurement systems was observed, mostly at MCP joint differences larger than about 10 deg. The PIP joint is more affected by the filter, and in a substantial portion of cases (83%) a significant improvement was observed. As indicated by the triangular markers on the horizontal axes in (Figure 2, graphs d2 to i4), no significant difference is present when the Nimble VR output overlap with the filter output, which occurs when the Nimble VR measurements already approach the true PIP joint angle. Moreover, at high standard deviations of Nimble VR data, statistically significant differences with the filter data are not always obtained.

Following, the separate hand postures will be discussed.

In the Flat Hand posture (Figure 2, graphs d_1_ to d_4_), with both MCP and PIP joint angles being 0 deg, the Nimble VR PIP joint estimate shows the poorest accuracy, especially at low hand pitch angles (graph d_2_). The Kalman filter output adjusts this and keeps both the MCP and PIP joint estimates around 0 deg, even in the ranges where hand orientation measurements are imprecise. This is most clearly shown by the decrease in standard deviation for both joints at graphs d_2_ to d_4_.

At Pure PIP Flexion (Figure 2, graphs e_1_ to e_4_), where the MCP joint angles are kept at 0 deg and the PIP joint at 90 deg, one can see large fluctuations of accuracy in the PIP flexion angle estimate. For the pitch range (graph e_2_), in the region below −50 deg, the PIP angle is grossly underestimated, but for the remainder of the range, it is close to the actual angle. The Kalman filter output decreases the large variations over this range, keeping the joint estimate relatively accurate with some fluctuations around the actual PIP angle. For both the yaw (graph e_3_) and roll (graph e_4_) orientation ranges an improvement in precision and less variation in the accuracy can be seen. The MCP joint estimate deviates from the actual angle at high pitch angles (graph e_2_), but is relatively accurate for the other orientations (graphs e_3_ and e_4_).

At Pure MCP Flexion, (Figure 2, graphs f_1_ to f_4_), with the PIP joint angles kept at 0 deg, a bias is seen during pitch (graph f_2_). As with pure PIP flexion, the PIP joint angle is wrongly estimated by the Nimble VR up until −10 deg, after which it correctly approaches the actual angle. The Kalman filter output adequately corrects for this bias, and keeps the estimated PIP joint angle around 0 deg at all angles. This comes, however, at the expense of the accuracy with which the MCP joint angle is estimated, which slightly worsens due to the Kalman filter. This is exemplified by the mean and standard deviations of the MCP joint angles taken over the entire range (see top left of graph f_2_), showing a slight increase in angle underestimation (*i.e.*, a lower accuracy) and an increase in standard deviation (*i.e*., a lower precision). However, the reverse is thus true for the PIP joint. This same effect is seen to a lesser extent for the yaw and roll orientations (graphs f_3_ and f_4_).

For Combined MCP and PIP Flexion*,* (Figure 2 graphs g_1_ to g_4_), which is a more natural hand closure posture than the pure flexion of either the MCP or PIP joints, the advantage of using the Kalman filter is most pronounced in the PIP joint estimate. Where the Nimble VR measurements for this joint greatly vary for all orientations and are grossly overestimated, the Kalman filter yields a reliable and more accurate PIP joint angle estimate.

Lastly, for the Pinch Grip posture (Figure 2, graphs h and i), we show both the index (graphs h_1_ to h_4_) and thumb fingers (graphs i_1_ to i_4_). Again, the Kalman filter increases the precision of the PIP joint output of the index finger (graphs h_2_ to h_4_). However, there is a significant overestimation of the joint angle for all orientations. For the thumb (graphs i_2_ to i_4_), the Kalman filter slightly increases precision for both joints estimates and slightly improves the MCP joint accuracy. However, the filter’s effect is less pronounced here as compared to the index finger.

### 4.3. Wooden Hand Model Finger Joint Measurements—All Fingers

**Figure 3:**
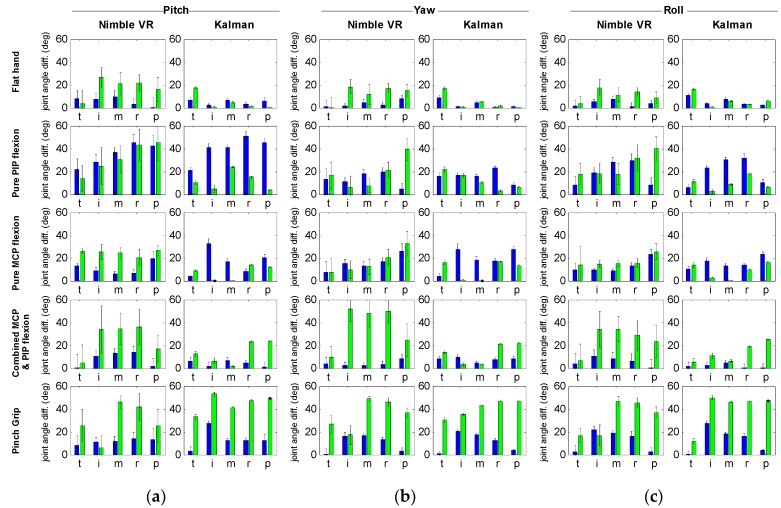
Precision and accuracy for all fingers of the hand model and for all orientation ranges. (**a**) Pitch range; (**b**) Yaw range; (**c**) Roll range. For each graph, the absolute difference is given between the mean calculated joint angle (*i.e.*, mean of the mean joint angles) and the true joint angle. The accompanying standard deviation is given as well (*i.e.*, the mean of the standard deviations at all angles), shown as ± 2·SD. Note that the values provided here are equal to the values given in the top left of the graphs in Figure 2 minus the true joint angles of the assessed hand pose. A distinction is made between the Nimble VR data (left) and the Kalman filtered date (right), as well as between the metacarpophalangeal (MCP, blue) and proximal interphalangeal (PIP, green) joint. From left to right the fingers are presented; t = thumb, i = index, m = middle, r = ring, p = pinky.

In order to assess the improvements gained for all fingers, in Figure 3 the difference between the true joint angles and the mean joint angles taken over the range of all assessed hand orientation ranges are given. These differences are equal to the mean joint angle in the top left of every graph in Figure 2 minus the true joint angle. The accompanying standard deviation is provided in Figure 3 as well. Hence, Figure 3 shows the mean accuracy and precision measures for all fingers, joints and poses, for the respective orientation range. It can be seen that for all fingers, the filter increases precision for the PIP joints and to a lesser extent for the MCP joints, regardless of hand posture. Accuracy improvements are seen for the PIP joints for the flat hand, pure PIP flexion, pure MCP flexion, and combined MCP and PIP flexion postures, but not for the pinch grip posture.

Lastly, we calculated the overall mean accuracy and precision improvements per joint gained by implementation of the filter. The overall accuracy and overall precision estimates were calculated across 2050 means and 2050 SDs, respectively (82 angles (19 pitch angles + 25 yaw angles + 38 roll angles) * 5 postures * 5 fingers). The results show that accuracy of the MCP slightly worsens by 6% from 12.7 deg (SD = 11.5 deg) to 13.5 deg (SD = 12.9 deg). This is offset by an accuracy improvement for the PIP joint of 31%, from 24.4 deg (SD = 17.4 deg) to 16.8 deg (SD = 15.7 deg). The precision of the MCP joint assessment improves with 5%, from 2.3 deg (SD = 2.5 deg) to 2.2 deg (SD = 2.2 deg), whereas the precision of the PIP joint improves with 79%, from 4.5 deg (SD = 4.1 deg) to 0.9 deg (SD = 1.1 deg). Overall, the filter thus marginally affects the MCP joint estimation, but strongly improves PIP joint estimation.

### 4.4. Human Hand Active Finger Flexion Measurements

**Figure 4:**
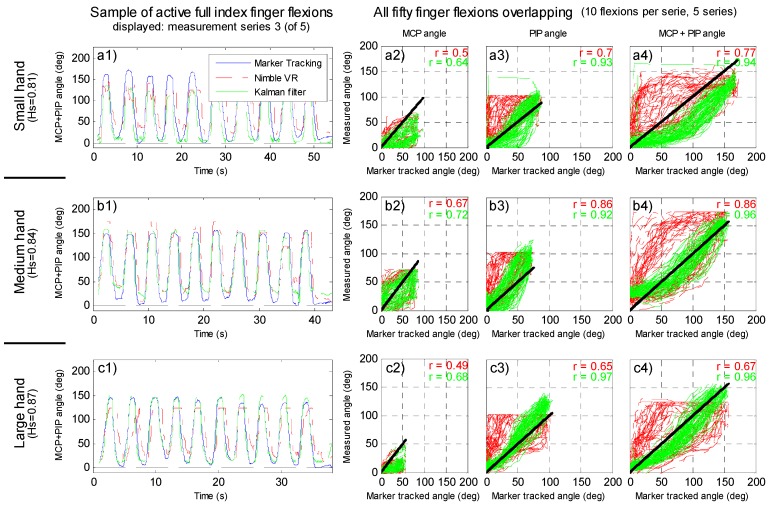
Finger joint estimates at dynamic finger flexions. (**a1–a4**) small hand size; (**b1–b4**) medium hand size; (**c1–c4**) and large hand size. Five sessions with 10 full finger flexion repetitions each were performed. All graphs show the Nimble VR system (red dash dotted line), Kalman filter output (green dashed line), and Marker Tracking measurements (blue continuous line). Left three graphs show the combined metacarpophalangeal (MCP) and proximal interphalangeal (PIP) joint angles of the third performed measurement session. The remaining nine plots show the Nimble VR and Kalman filter output plotted *vs.* the Marker Tracked angles of all fifty finger flexions performed per hand. The black line presents the true angle, and the plots are given for separate and combined joints: (a2–c2) MCP; (a3–c3) PIP; (a4–c4) combined MCP plus PIP joints. In the top right, the Pearson correlation coefficient between the measured angle and marker tracked angle are given (red = Nimble VR, green = Kalman filter output).

**Figure 5:**
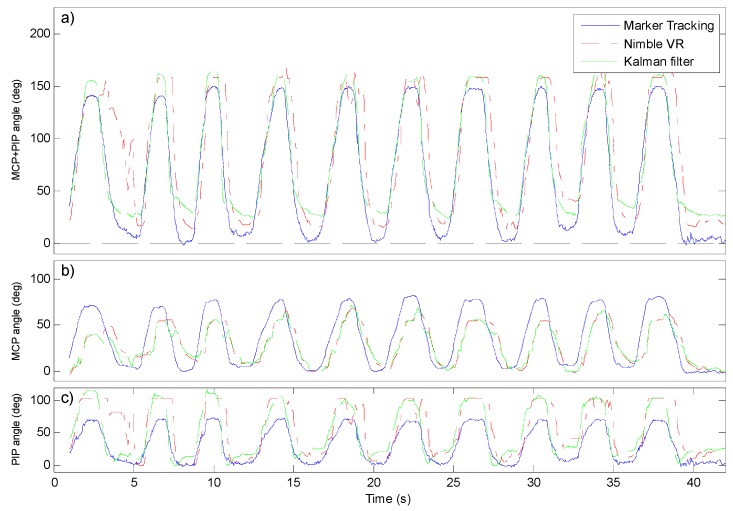
Active index finger flexion comparison between Marker Tracking, Nimble VR, and Kalman filtered joint angles. Shown data are from the medium sized hand, first measurement session. (**a**) sum of the metacarpophalangeal (MCP) and proximal interphalangeal (PIP) joint angles plotted versus time; (**b**) MCP joint angles versus time; (**c**) PIP joint angles versus time.

Dynamic flexing of the fingers while performing Marker Tracking of the joint angles and measuring the Nimble VR, Data Glove, and Kalman filter output yields the results shown in Figure 4 and Figure 5. In Figure 4, one of five sessions are shown, for the three differently sized hands. Additionally, all fifty full finger flexions per hand as measured with the Nimble VR system and obtained through the Kalman filter are plotted *vs.* the marker tracked angles. In Figure 5 the MCP and PIP joints are shown separately as well in combination for 10 flexions performed by the medium sized hand (Hs = 0.84). Note that the Marker Tracking results are free from any time delay.

In Figure 4, at the peaks of the graphs (*i.e.*, the points of maximum finger flexion), a noticeable effect can be seen of the hand size on the degree of under- or overestimation of the joint angles as compared to the Marker Tracking angles. The following presented under- or overestimation values have been calculated over all 50 flexions combined for each hand. At the small hand (graphs a1-a4) one can see that both the Nimble VR and the filter output underestimate the MCP joint angle considerably by 51 deg. However, this is compensated by an overestimation for the PIP joint (Nimble VR: 13 deg, SD = 17 deg; Kalman filter: 14 deg, SD = 11 deg), resulting in an overall underestimation of the full finger flexion by 38 deg (SD = 12 deg) with the Nimble VR system and 36 deg (SD = 8 deg) for the Kalman filter output. This underestimation of the MCP joint angle is less prominent at the medium sized hand (graphs b1-b4), where the Nimble VR underestimates the MCP joint with 15 deg (SD = 15 deg), overestimates the PIP joint with 28 deg (SD = 8 deg), leading to an overall overestimation of 13 deg (SD = 16 deg). For the medium sized hand, the output from the Kalman filter underestimates the MCP joint with 16 deg (SD = 15 deg) and overestimates the PIP joint with: 26 deg (SD =10 deg), adding up to a combined overestimation of 9 deg (SD = 9 deg). Lastly, for the large hand (graphs c1-c4), an underestimation is again seen for the MCP joint (filter: 23 deg, SD = 11 deg), but the filter output overestimates the PIP joint (filter: 27 deg, SD = 9 deg), leading to an overall small overestimation of 4 deg (SD = 12 deg) (as compared to the Nimble VR output providing an underestimation of 29 deg, SD = 20 deg).

Summarizing, the Kalman filter system underestimates the MCP joint angle (small, medium, large hand underestimation: 61%, 20%, and 50%, respectively) while the PIP joint is overestimated (small, medium, large hand overestimation: 18%, 34% and 28%, respectively). The combined finger flexion approximation is underestimated at the small hand (small hand: 22%), but marginally overestimated at medium and large hands (medium hand: 6%; large hand: 3%). The overall contribution of the Kalman filter compared to the Nimble VR data is relatively limited for the small and medium sized hands, providing a 1% and 3% reduction in under- and overestimation respectively. However, at the large hand Nimble VR data an underestimation of 20% is present, which after implementation of the filter changes to a small overestimation of 3%.

The correlation coefficients provided in the top right of the graphs in Figure 4 indicate the degree of linearity in the datasets. At all hands and finger joints, a higher correlation coefficient was found in the Kalman filter output than for the Nimble VR. The correlation for the Kalman filter output is relatively low at the MCP joint of the small hand (*r* = 0.64), and strongest at the PIP joint of the large hand (*r* = 0.97).

The root-mean-square errors (RMSE) calculated of the Nimble VR and Kalman filter output with respect to the marker tracked angles for the small hand were 43 deg and 33 deg, for the medium hand 38 deg and 20 deg, and the large hand 39 deg and 16 deg, respectively. A substantial improvement is thus visible when using the Kalman filter. Important to note is that these RMSE values were calculated over the entire 50 hand flexures, where the maximum absolute error at a particular point in time was 167 deg for the Nimble VR system, and 158 deg for the Kalman filter.

Lastly, in Figure 5, the discrepancy in time between the Nimble VR angle measurement and the Marker Tracking output is shown. A delay is present at both joints. Although for the Kalman filter output at the MCP joint the delay of 0.4 s persists (SD = 0.2 s), calculated over the 50 finger flexions of the medium sized hand, this effect is less pronounced at the PIP joint. For the PIP joint, the delay before and after implementation of the filter are 0.17 s (SD = 0.07 s) and 0.07 s (SD = 0.04 s), respectively. The resulting combined finger flexion estimation has a delay of 0.23 s (SD = 0.07 s) before implementation of the filter, and 0.12 s (SD = 0.03 s) after. Lastly, looking at the Nimble VR data at the PIP joint one can see that this joint is at times measured unsteadily, and that some erratic fluctuations occur, which are smoothed in the filter output.

## 5. Discussion

The results showed that the application of the Kalman filter for fusing vision based with contact based tracking data provided substantial improvements in precision, and to a lesser extent improvements in accuracy. Using the Data Glove improved hand posture and finger flexion estimates, especially at the occurrence of visual self-occlusion of the fingers. Depending on the orientation and posture of the hand, these precision and accuracy improvements varied somewhat.

### 5.1. Setup Limitations

The measurements of the Nimble VR is influenced by the chosen depth camera and its resolution. Aside from this, two limitations were present in our setup: (1) the anatomical dissimilarities of the wooden hand model with respect to real hands; and (2) the sensor limitations of the Data Glove.

The first limitation was the fact that we used a wooden hand model. Although the hand model was anatomically correct in terms of dimensions and locations of the MCP, PIP and DIP joints of the fingers, it is less representative for the thumb. The model lacks the carpometacarpal (CMC) joint of the thumb, which connects the first metacarpal bone of the thumb to the carpal bone of the wrist. This joint, allowing for about 55° of flexion and 10° of hyperextension, is important for the action of opposition (*i.e.*, touching the tip of the pinky finger with the tip of the thumb). Due to the absence of the CMC joint, both palmar and radial abduction are impossible in the hand model, limiting the thumb’s movements to flexion of the MCP and IP joints. As a result, the pinch grip pose that we assessed deviated from an actual pinch grip that relies on the CMC joint. The Nimble VR software (which compares the obtained Kinect camera output to a precomputed database of hand postures) is able to detect this pinch grip posture, and automatically assumes the CMC joint to play a role in it. As a result, the software output better reflects real index and thumb fingers joint angles as compared to the model fingers. If the reader was to place one’s own hand in the pinch grip pose, and compare this to the hand model pinch grip (shown in Figure 2 graphs h_1_ and i_1_), he/she can see the degree of flexion of the MCP joint of the index finger to be nonzero and PIP joint to be around 8°, whereas the model had 0 and 50° respectively. When calculating the accuracy of the MCP and PIP joints over all hand poses combined, but excluding the pinch grip pose, the Nimble VR provides 12.8 deg and 22.1 deg, respectively, and after implementation of the filter this is 13.4 deg and 10.5 deg. Compared to previous results, the accuracy for the PIP joint is thus improved by 6 deg when not taking the pinch grip into consideration. The precision stays approximately the same.

An additional restriction of the hand model is that its fingers are not able to perform abduction and adduction at the MCP joints. This did not affect our measurements of the MCP and PIP joints, but limited us in the selection of the postures. For future research, it is interesting to use a hand model that is able to make such joint movements, and to use a Data Glove with additional sensors that measure ab- and adduction of the fingers, such as the 5DT Data Glove 14 Ultra [52]. However, a glove with more sensors is more likely to impede the naturalness of motion of the user, especially considering the fact that ab- and adduction sensors need to be placed in between the fingers.

The second limitation of the used measurement setup was inherent in the Data Glove. Because the glove itself is made of stretch Lycra and made to fit most hand sizes, its measurement accuracy is dependent on the quality with which it is calibrated. Furthermore, its fibre-optics-based bending sensors are positioned inside small hollow sleeves along the glove fingers. Consequently, the sensors are slightly able to shift inside these sleeves, potentially creating drift in the measurements during prolonged usage. As the measurements presented in this research were acquired during passive hand postures, this drift could not be quantified.

Another disadvantage of using the Data Glove is that the predetermined Data Glove weights $w_{DG_{MCP}}$ and $w_{DG_{\mathrm{PIP}}}$ (see Appendix C) are person-specific to a certain extent. As the size of peoples’ hands vary, the degree of sensor flexion and their exact positioning with respect to the fingers tend to vary as well. The current weights were determined based on a medium sized hand. Where for example the sensor on the pinky mainly measures PIP flexion, this may be slightly different for persons with smaller hands, where the sensor will overlay more of the MCP joint. Based on the active finger flexion measurements performed on hands of different sizes, we found that the degree of overall finger flexion is underestimated at small hands, and slightly overestimated at medium and large hands. As such, the glove weights will likely not have to be recalibrated for all test participants. In order to improve the joint angle estimations for small hands, a second set of glove weights may be measured and used in future tests.

### 5.2. Active Finger Flexion Measurements

When assessing the influence of the Kalman filter on the active finger flexions (Figure 4), it can be seen that the time delay inherent in the Nimble VR remains present at the MCP joint estimate, but is reduced at the PIP joint. Similarly, for all the fingers (Figure 3), the MCP joint’s increase in precision and accuracy is not as pronounced as for the PIP joint. The explanation for this can be found in the implementation of the variance matrix $R_{k}$ (see Appendix A). The observed variance of the Nimble VR PIP joint angles is higher as compared to the MCP joints. The filter therefore assumes the Nimble VR MCP joint estimates to be more reliable than those of the PIP joint. As a result, the Data Glove measurements are predominantly used to smoothen out and correct the PIP joint estimates.

During active finger flexions, both the MCP and PIP joints are flexed simultaneously. It is likely that for the Kinect camera, which has a top down view of the hand, the flexion of the PIP joints is initially visually more pronounced than the flexion of the MCP joint. The flexion of the MCP joint is not detected directly, providing an explanation for the time delay (which, as shown in Figure 5, is larger for the MCP joint compared to the PIP joint). As the Data Glove mostly corrects for the PIP joints, this time delay persists in the filter output for the MCP joints. In order to correct for this, one could adjust the variance terms in matrix $R_{k}$ accordingly, allowing the Data Glove measurements to better influence the MCP joint estimates. However, this would come at the cost of the quality of the PIP joint estimates.

The size of the time delays for both joints can be further reduced, as the setup in its current iteration has not yet been optimized in terms of computational efficiency. The time delays have been obtained on a computer with an i7-2640M CPU (2.80 GHz), 8 GB of RAM and 64-bit Windows 7 Operating System, while using custom written software to capture the data stream from both the camera and data glove system. The time delays will likely decrease with further iterations of the Nimble VR software as well as through implementation of a dedicated processing unit to perform the relatively heavy Nimble VR calculations.

### 5.3. Data Fusion Improvements

Sensor redundancy through data fusion of a contact based device with that of a vision based provides data completeness during partial and full visual occlusion of the fingers. Although the Kalman filter method is easy in implementation, it by definition requires the state prediction model to be a linear function of the measurements. It is possible that an extended Kalman filter, using non-linear functions, will provide more accurate state estimates. An interesting way to extend the model could be to use heuristics, and to change the model based on the hand pose detected by the Nimble VR software. For example, if a person is pointing with the index finger, one could assume him or her to have the remaining fingers closed towards the palm of the hand. As such, it is known there is a high likelihood that some of those fingers will be visually occluded, and the model could take this into account.

Another way of improving the current setup may be to use alternative contact based measurement devices that directly measure finger flexion without impeding naturalness of motion of the user. Although the used 5DT data glove is not very intrusive, studies have shown that gloves may have negative effects on manual dexterity, comfort, and possibly the range of finger and wrist movements [58]. One interesting method presented in literature is the use of adhesives to attach sensor sleeves to the back of the fingers whilst leaving the joints free of adhesives that would restrict movements [59]. These sensors would leave the fingers largely unimpeded, benefiting potential uses for tactile feedback.

An alternative may also be to exchange the contact based device for a locally functioning vision based device such as the Digits wrist-worn gloveless sensor [60]. Digits is a small camera-based sensor attached to the wrist that images a large part of the user’s bare hand. Its infrared camera is placed such that the upper part of the palm and fingers are imaged as they bend inwards towards the device. As the device is not restricted to a fixed space around the user, but moves with the user’s hand, it can image the fingers that may be occluded from vision for the Nimble VR.

A third potential device for integration with the Nimble VR system is the Myo armband from Thalmic Labs [61,62]. Instead of approximating the flexion of the fingers, this system extracts user gestures from measured EMG signals. Using these observed gestures to update the Nimble VR’s calculated skeletal model may be a different route for obtaining improved finger joint angles estimates.

Regardless of which second measurement system is used to improve data completeness through data fusion, in case of a contact based system its influence on the naturalness of motion needs to be taken into account. Especially in applications such as laparoscopic training, such (minor) physical limitations can easily become a hindrance to the participants (or surgeons), and influence task performance. The advantages of more complex measurement systems should thus be considered within the context and duration of the target application(s).

The Nimble VR software and concurrent hardware is still under development, as the company Nimble VR has recently joined Oculus VR, LLC [38]. It is expected that the accuracy and precision will improve prior to consumer market introduction. Additionally, especially with respect to the consumer market, the advantages of fusing simple unobtrusive contact based sensors with “low-budget” vision based systems (e.g., Leap Motion and Nimble VR) may be an easy and computationally efficient way of obtaining data completeness for applications such as 3D computer interaction and gaming [63].

### 5.4. Application in Medical Field

One of the fields where precision and data completeness of measured hand and finger motions is of prime importance is the medical domain. As described in the introduction, hand motions and postures as HCI input have already been applied in several cases [10,11], but not yet in virtual laparoscopic training. The Nimble VR system used in this research has a working area large enough to allow surgeons to make the same surgical routine motions they would make using the already available Da Vinci master robot (Intuitive Surgical Inc., Sunnyvale, CA, USA) to which some surgeons have already grown accustomed [47,48]. Before the eventual implementation of vision based devices in applications such as laparoscopic training however, several limitations will need to be overcome. Foremost is the time delay in the detection of the hands by the camera and the (computationally heavy) extraction of the skeletal model of the hand. A time delay of 300 ms is likely to aversely effect skill training [64]. In terms of surgical performance, it has been shown that surgeons are able to compensate for time delays up to 700 ms through a “move and wait” strategy. However, the number of operator errors increases with increasing time delay [65,66,67]. Hence, ideally the time delay has to be minimized. Secondly, inherent in the use of a vision based device is the lack of haptic feedback, a property which has been shown to improve hand–eye coordination [68]. To certain extents, this limitation can be addressed through the use of visual force feedback [69], implementation of pseudo-haptic feedback in the virtual environment [70], or the integration of a haptic-feedback mechanism in a touch based glove [71].

The precision of the joint angle estimates obtained through implementation of the data glove and the Kalman Filter in this research is generally around 1 to 3 deg, depending on which joint and finger we are looking at. For medical practice, a control precision of 2 mm for standard laparoscopic instruments has been reported [72]. As an example, when controlling a joint incorporated in the shaft of a laparoscopic instrument, depending on the length of the segment attached to the joint controlled through finger flexion, a standard deviation from 1 to 3 deg is acceptable (e.g., with a segment length of 20 mm, which is the approximate length of a functional tooltip of a laparoscopic instrument, an angular standard deviation of 3 deg equals a tip positional standard deviation of 1.0 mm).

The accuracy improvements through implementation of the filter were less pronounced as compared to the precision results. Using the filter, for the MCP joint, the mean deviation from the true joint angle was 13.5 deg, and for the PIP joint around 16.8 deg. Due to the good precision however, touchless control of simulated instruments through flexion of the fingers combined with movements of the hand and concomitant visual feedback should be possible. This is true when considering that humans are able to use visual feedback to correct for unforeseen perturbations during continuous hand movements [73]. Therefore, considering the precision of the joint angle estimates obtained in this research, our aim is to implement and further study the presented measurement setup for VR medical simulator purposes.

## 6. Conclusions

In this study, we performed a Kalman filter data fusion of hand and finger motion measurements obtained with the 5DT Data Glove and the Nimble VR using a Kinect camera. Measurements were obtained using a wooden hand model placed in various postures across various orientation ranges, as well as on three different sized hands performing active finger flexions. Through sensor redundancy, more accurate and substantially more precise joint flexion estimates could be obtained compared to the Nimble VR alone. The obtained accuracy for the MCP and PIP joint after implementation of the filter were 13.5 deg (SD = 12.9 deg) and 16.8 deg (SD = 15.7 deg), respectively, and the precisions, 2.2 deg (SD = 2.2 deg) and 0.9 deg (SD = 1.1 deg). Thus, for the PIP joint, a 31% improvement in accuracy was observed and a 79% improvement in accuracy. The MCP accuracy worsened by 6% and the precision improved by 5%, showing the filter to only marginally influence this joint. Due to the use of the contact based Data Glove, visual self-occlusion of the fingers for the visual based Nimble VR system could be mitigated, and data completeness obtained.

## Figures and Tables

**Figure A1:**
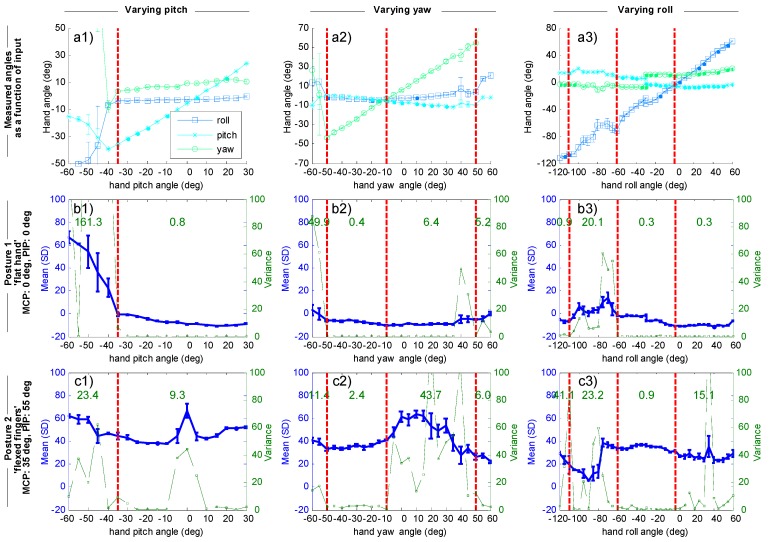
(**a1–a3**) Measured hand orientations; (**b1–c3**) metacarpophalangeal (MCP) index finger joint angle as a function of hand orientations (pitch, roll and yaw) for two different postures. (**b1–b3**) posture 1, flat hand; (**c1–c3**) posture 2, flexed fingers. Means, standard deviations, and variances were calculated for each of the 5 measurement sessions, and subsequently averaged over all sessions. Posture 1 is with a “flat hand”, MCP 0 deg and proximal interphalangeal (PIP) 0 deg joint angles; posture 2 is with flexed fingers, MCP 35 deg, PIP 55 deg. The thick (blue) line are the measured joint angles. At posture 1 (with MCP 0 deg) one would expect the measurement data as a function of changing hand orientation to be stable around 0 deg, and at posture 2 (with MCP 35 deg) stable around 35 deg. The dashed (green) lines are the measured variances. Vertical thick dashed lines (red) distinguish between stable and unstable orientation ranges, and the therein provided numbers are the mean variances for those ranges.

**Figure B1:**
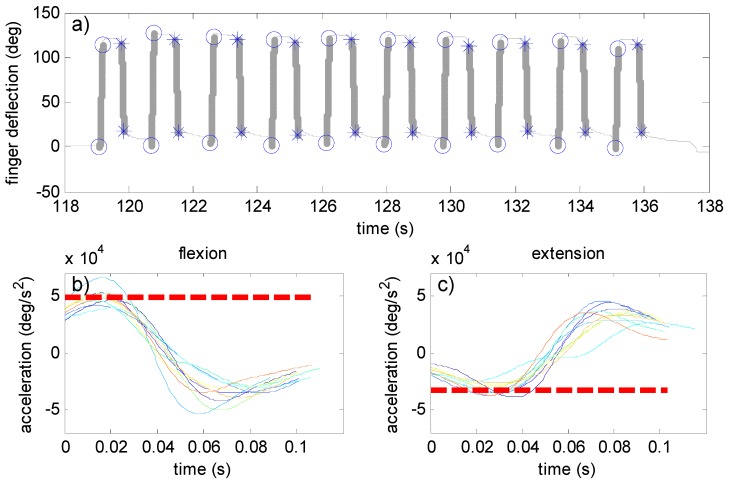
**Top**: (**a**) Index finger motion measured with 5DT Data Glove during 10 times as-fast-as-possible finger flexions of a single participant. Indicated with a bold line are the flexion and extension movements with their respective marker indicators (o and *) showing starting and stopping points of the finger motions; (**b**) calculated accelerations over the course of every finger flexion superimposed over each other; (**c**) similar as subfigure b, but for finger extensions. The horizontal (red) dotted lines show the mean peak accelerations determined from the datasets. (Note: this participant was faster than average.)

**Figure B2:**
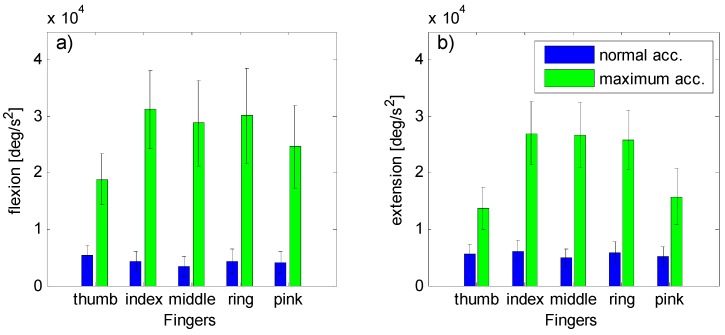
Mean peak accelerations (±SD) during finger flexion and extension performed at normal speed (blue) and as fast as possible (green), calculated per participant and subsequently averaged over all 10 participants. (**a**) finger flexion measurements; (**b**) finger extension measurements.

**Figure C1:**
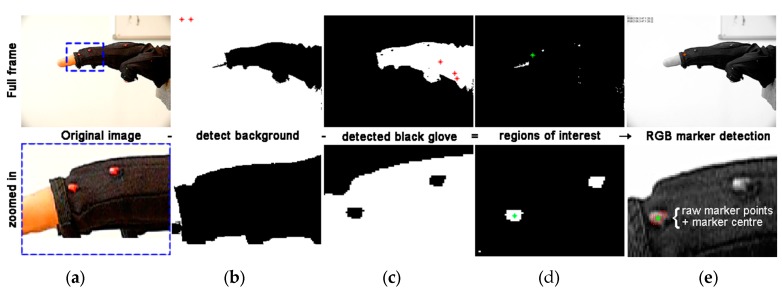
Marker Tracking video analyses. Example of one analyzed frame for the tracking of a single marker. From left to right: (**a**) original frame; (**b**) detected background in white, residual image information in black; (**c**) detected black glove where the black dots inside the hand show the edges of the markers; (**d**) residual image information after background and glove subtraction; and (**e**) red marker pixels detected from 4 overlaid on original image and mean shift cluster detection used to determine the center of the marker (shown with green + in image).

**Figure C2:**
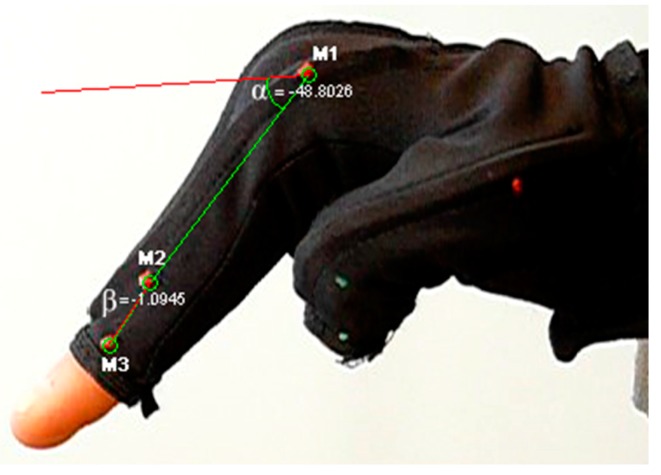
Example of detected metacarpophalangeal joint (MCP, α) and proximal interphalangeal (PIP, β) angles as a function of detected marker locations. Angles provided in degrees.

**Figure C3:**
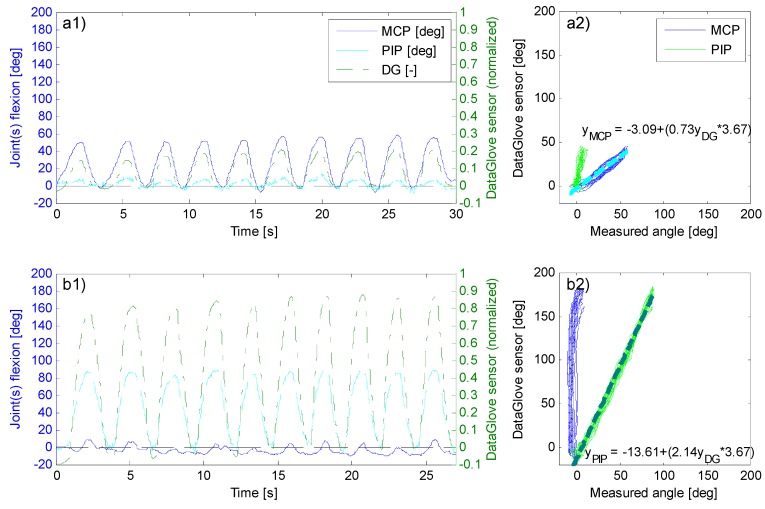
(**a1–b1**) Raw metacarpophalangeal (MCP, **a1**) and proximal interphalangeal (PIP, **b1**) joint measurement data collected through Marker Tracking and normalized Data Glove (DG) sensor readings for the index finger; (**a2–b2**) Joint flexions measured through Marker Tracking versus data glove sensor readings rescaled to range [0 210]. The gradient of the linear least-squares fit function provides the weights $w_{DG_{MCP}}$ and $w_{DG_{PIP}}$.

## Appendix A

### Orientation Dependent Variance Quantification

#### Methods

Finger joint variances for matrix $R_{k}$ were measured using an anatomically correct wooden right-hand model mounted on a tripod with a 3-way pan/tilt head. The wooden hand had a length of 21.35 cm, measured from wrist to tip of the middle finger, and breadth of 8.3 cm.

Two different hand postures were assessed: (1) hand with the fingers held stretched (“flat hand”), and (2) bend fingers (with index through pinky joint angles 35° MCP and 55° PIP). The orientation of the hand model was varied by placing it in varying pitch, roll and yaw angles (ranges [–60, 30] deg, [–120, 60] deg and [–60, 60] deg, respectively). All three angles were varied at 5 deg intervals, while keeping the other two angles constant at 0 deg.

At every step, 200 samples were taken in approximately 13 s. Every measurement session was repeated five times. For every combined session, the mean and variance were determined of the orientation of the hand and the angular measured MCP and PIP joints rotations. Based on these measurements, the variances as a function of changing hand orientation and posture were determined.

#### Results

Figure A1 provides an overview of the measured hand orientation and the MCP joint angles of the index finger for varying hand orientation angles and both evaluated hand postures. In these figures, ranges are indicated using vertical (red) dotted lines. These ranges correspond to stable or unstable finger flexion measurements.

For varying pitch angles, and independent of posture, the MCP and PIP joints can adequately be estimated in the range [−35, 30] deg. In this stable range, at hand posture 1 (“flat hand”) no substantial difference is present between the MCP and PIP joints. However, at posture 2 (finger flexion) the PIP joints are subject to higher variances than the MCP joints (75.9 deg^2^ *vs.* 9.3 deg^2^, respectively). No apparent relation is present however between variance and pitch angle. At hand orientation angles below −35 deg, the hand is angling too far downwards for the camera to robustly detect the joint angles. Lastly, for posture 2, the PIP joints appear to be overestimated.

The yaw angles are measured robustly in range [−50, 50] deg. Outside this range, the measurement results are unstable. The yaw stable range can be divided into two separate ranges, depending on whether the thumb is pointing towards or away from the computer screen. These ranges are [−50, −10] deg and [−10, 50] deg, respectively, with measured MCP variances 2.4 deg^2^ and 43.7 deg^2^ for the index finger at posture 2. We thus measure a higher variance when the thumb is pointing away from the computer screen. The PIP flexions are overestimated at posture 2.

Lastly, the roll angle measurements are stable over the entire range [−120, 60] deg, except for the range [−110, −60] deg where uncertainty is present in the data. At −90 deg, the hand is angled vertically, with the thumb facing up, and the middle through pinky fingers are occluded for the camera by the thumb and index fingers. As a result, finger joint estimates are unstable in this range, and have higher variance as compared to the stable range (for the MCP joint 20.1 deg^2^ *vs.* 0.3 deg^2^ at posture 1 and 23.2 deg^2^ *vs.* 8.1 deg^2^ at posture 2). At range [40, 60] deg, when the thumb is pointing down, the software has significant difficulty detecting the thumb (resulting in either high or zero variance for the thumb). In this range, at high thumb measurement variance, all the other fingers are influenced as well, explaining the variance peak at posture 2 at 40 deg, see Figure A1. Again, in the stable range ([–120, –110] deg and [–60, 60] deg), the PIP variance is higher at posture 2 due to camera occlusion as compared to posture 1. This PIP variance is also higher in range [0, 60] deg, when the thumb is pointing down, as compared to [–60, 0] deg (65.4 deg^2^ *vs.* 17.3 deg^2^ for the PIP joint, respectively, at posture 2).

Based on these results, the variances of individual fingers and joints can be expressed as a function of hand orientation and degree of finger flexion. Where possible, the measured orientation is used as input in choosing the appropriate corresponding variance in matrix $R_{k}$. Moreover, where higher variance is measured at posture 2, the normalized measurement signal from the Data Glove is used as a weight in scaling up the joint Posture 2, the normalized measurement signal from the Data Glove is used as a weight in scaling up the joint variance at increasing finger flexion. Within the stable orientation ranges (pitch α [−35, 30] deg, roll β [−120, 60] deg, yaw γ [−50, 50] deg) the parameters for matrix $R_{k}$ for the MCP joint of the index finger are calculated as follows:

(A1) $$\sigma_{NVR_{MCP}}^{2}\left(\alpha ,\beta ,\gamma \right)=A+B\cdot z_{DG}=\max\left[\begin{array}{c} \sigma_{NVR_{MCP}}^{2}\left(\alpha \right)\\ \sigma_{NVR_{MCP}}^{2}\left(\beta \right)\\ \sigma_{NVR_{MCP}}^{2}\left(\beta \right) \end{array}\right]\text{, with}\;\begin{array}{cccc} \sigma_{NVR_{MCP}}^{2}\left(\alpha \right)= & 0.8+8.5\cdot z_{DG} & \text{if} & -35\leq \alpha \leq 30\\ \sigma_{NVR_{MCP}}^{2}\left(\beta \right)= & \left\{ \begin{array}{l} 0.9+41.1\cdot z_{DG}\\ 23.2\\ 0.9\\ 0.3+14.9\cdot z_{DG} \end{array}\right. & \begin{array}{l} \text{if}\\ \text{if}\\ \text{if}\\ \text{if} \end{array} & \begin{array}{c} -120\leq \beta \leq -110\\ -110<\beta \leq -60\\ -60<\beta \leq 0\\ 0<\beta \leq 60 \end{array}\\ \sigma_{NVR_{MCP}}^{2}\left(\gamma \right)= & \left\{ \begin{array}{l} 0.4+2.0\cdot z_{DG}\\ 6.4+37.3\cdot z_{DG} \end{array}\right. & \begin{array}{c} \text{if}\\ \text{if} \end{array} & \begin{array}{c} -50\leq \gamma \leq -10\\ -10<\gamma \leq 50 \end{array} \end{array}$$

The *A* and *B* values (with unit deg2) for varying orientation angles for all fingers have been determined separately, and have been given below in the format $\left[\begin{array}{cc} A_{MCP} & B_{MCP} \end{array}|\begin{array}{cc} A_{PIP} & B_{PIP} \end{array}\right]$ for each finger.

(A2) $$\begin{array}{ccccc} \mathrm{thumb} & \mathrm{index} & \mathrm{middle} & \mathrm{ring} & \mathrm{pink}\\ \left[\begin{array}{cc} 3.7 & 37.5\\ 0.1 & 5.9\\ 21.8 & 0\\ 1.9 & 0\\ 2.4 & 23.3\\ 8.1 & 0\\ 19.1 & 23.3 \end{array}|\begin{array}{cc} 11.7 & 62.4\\ 3.5 & 7.8\\ 15.9 & 0\\ 6.4 & 0\\ 5.5 & 16.2\\ 19.6 & 0\\ 33.8 & 45.1 \end{array}\right] & \left[\begin{array}{cc} 0.8 & 8.5\\ 0.9 & 41.1\\ 23.2 & 0\\ 0.9 & 0\\ 0.3 & 14.9\\ 0.4 & 2.0\\ 6.4 & 37.3 \end{array}|\begin{array}{cc} 2.3 & 73.6\\ 2.8 & 85.3\\ 41.2 & 0\\ 0.7 & 16.6\\ 0.7 & 55.7\\ 0.6 & 25.0\\ 11.5 & 214.8 \end{array}\right] & \left[\begin{array}{cc} 0.8 & 11.6\\ 0.5 & 19.1\\ 16.6 & 0\\ 1.3 & 0\\ 0.3 & 14.8\\ 0.3 & 2.0\\ 5.8 & 43.7 \end{array}|\begin{array}{cc} 2.0 & 58.1\\ 0.7 & 26.9\\ 19.7 & 0\\ 0.4 & 41.1\\ 0.2 & 95.1\\ 0.2 & 33.8\\ 29.2 & 143.9 \end{array}\right] & \left[\begin{array}{cc} 0.9 & 32.4\\ 0.5 & 31.7\\ 26.9 & 0\\ 0.7 & 0\\ 0.4 & 11.7\\ 0.4 & 1.0\\ 4.0 & 75.3 \end{array}|\begin{array}{cc} 1.5 & 61.2\\ 1.8 & 103.1\\ 22.5 & 72.8\\ 1.0 & 14.6\\ 1.5 & 44.2\\ 0.8 & 28.3\\ 36.5 & 80 \end{array}\right] & \left[\begin{array}{cc} 1.4 & 66\\ 2.4 & 83.1\\ 9.3 & 48.6\\ 1.1 & 2.9\\ 1.1 & 6.6\\ 0.9 & 0.9\\ 7.8 & 70.4 \end{array}|\begin{array}{cc} 2.0 & 38.4\\ 4.7 & 58.7\\ 47.0 & 25.7\\ 2.6 & 27.9\\ 1.5 & 27.8\\ 2.9 & 19.6\\ 39.3 & 88.2 \end{array}\right] \end{array}$$

## Appendix B

### Fingers Maximum Acceleration Determination

#### Methods

The process noise covariance matrix $Q_{k}$ is calculated based on the maximum possible accelerations of the MCP and PIP joints. Measurements of these accelerations were performed using the 5DT Data Glove. Ten healthy young participants were asked to flex and extend their fingers at a normal pace ten times, and as fast as possible ten times. The measurement frequency of the Data Glove was 200 Hz. Measured flexion data were resampled to 1000 Hz, using a cubic interpolation method, and filtered using a 2nd order Butterworth filter with a cut-off frequency of 10 Hz. At both movement tasks, for each participant the peak accelerations at every flexion and extension for every finger were determined, and the mean (±standard deviation (SD)) calculated. All determined mean accelerations and SD were subsequently averaged over all participants, and we use this as input for matrix $Q_{k}$.

#### Results

An example of a single measurement session for the index finger is shown in Figure B1, with accompanying calculated finger flexion and extension accelerations. The participant was asked to flex his fingers as fast as possible. Accelerations and standard deviations averaged over all 10 participants are shown in Figure B2. The accelerations of the index, middle and ring fingers lie in the range 2.5 to 3.2·10^5^ deg/s^2^, and accelerations of the thumb and pinky are generally lower.

In order to make a distinction between ${\ddot{\varphi }}_{MCP}$ and ${\ddot{\varphi }}_{PIP}$, we use the weights calculated in Appendix C that indicate the individual joint contributions to the overall finger flexion angle as measured with the Data Glove. Recalculating these weights, which are given in equation (C.2), to percentages gives a MCP vs PIP division of 44% *vs.* 56% for the thumb, around 25% *vs.* 75% for the index, middle and ring finger, and 6% *vs.* 94% for the pinky. The MCP angle contribution to the Data Glove sensor readings is always lower as compared to the PIP angle due to shifting of the sensors in the glove, and the MCP joint angle being more gradual as compared to the PIP joint angle.

For matrix $Q_{k}$ we take the PIP joint contribution percentages and calculate the maximum joint accelerations by taking the same percentage from the measured overall flexure acceleration. For the index finger, with a total acceleration of 3.1·10^4^ deg/s^2^ we thus find a PIP joint acceleration of 0.75 * 3.1·10^4^ = 2.3·10^4^ deg/s^2^. The following accelerations were found for the respective fingers:

(B1) $$\begin{array}{cccccc} & \mathrm{thumb} & \mathrm{index} & \mathrm{middle} & \mathrm{ring} & \mathrm{pink}\\ {\ddot{\varphi }}_{PIP}= & [1.05 & 2.34 & 2.10 & 2.38 & 2.31] \end{array}\begin{array}{c} \cdot 10^{4}\;deg/s^{2} \end{array}$$

Because of the large spread in maximum accelerations (see Figure B2) between participants, these results need to be interpreted with caution. For implementation into the Kalman filter we will assume the MCP joint accelerations to be on par with the PIP accelerations, *i.e.*, ${\ddot{\varphi }}_{PIP}\approx {\ddot{\varphi }}_{MCP}.$ Moreover, considering that the accelerations are the maximum possible, and the normal movement accelerations are generally a lot lower, we can downscale the in (B.1) given values for input into $Q_{k}$ to increase joint angle approximation precision under the assumption of normal task operation speeds.

## Appendix C

### Determination of Data Glove weights

Data Glove weights $w_{DG_{MCP}}$ and $w_{DG_{\mathrm{PIP}}}$ were calculated based on reference measurements performed through colored Marker Tracking (MT) of the MCP and PIP joint rotations. The weights were calculated as follows:

(C1) $$\begin{array}{l} w_{DG_{MCP}}=\frac{\varphi_{DG_{MCP+PIP}}}{\varphi_{MT_{MCP}}}\text{, at pure MCP flexion }\left(\varphi_{PIP}=0\right)\\ w_{DG_{PIP}}=\frac{\varphi_{DG_{MCP+PIP}}}{\varphi_{MT_{PIP}}}\text{, at pure PIP flexion }\left(\varphi_{MCP}=0\right) \end{array}$$

where $\varphi_{MT_{MCP}}$ and $\varphi_{MT_{PIP}}$ are the joint angles as measured through Marker Tracking and $\varphi_{DG_{MCP+PIP}}$ the MCP and PIP combined angle calculated from the measured Data Glove sensor measurements. As the functions describe, by flexing for example the MCP joint (and consciously keeping PIP joint flexion as close to zero as possible), the ratio between the Data Glove measurement and the actual finger joint angle was determined. Note that consciously keeping one joint unflexed whilst flexing the other is physically challenging, hence the weights calculated from actual measurements need to be interpreted as approximations. In the case of an obtained value lower than one, the Data Glove has a bias towards joint angle under estimation and vice-versa.

Measurements were performed on the hand of the author, with colored markers attached to the joint locations of the index, pinky, and thumb fingers of the Data Glove. The MCP and PIP joints of the index through pinky fingers were flexed and extended separately at a relaxed pace, with the hand in view of the camera. The RGB data of the video footage was then analyzed for every movie frame (with 30 frames per second) through subtraction of the background and the black glove, followed by marker detection using a RGB threshold and Mean Shift Cluster detection (“Mean Shift Clustering” function code available at MATLAB Central) [57]. Analyses of a single frame are depicted in Figure C1. Drawing straight lines between the marker locations, and calculating the relative angles between those lines yielded the MCP and PIP joint angles (Figure C2). The measured joint angle through Marker Tracking were plotted *vs.* the measured Data Glove joint angles rescaled to range [0 210] deg, see Figure C3, right plots. This range is equal to the sum of the natural MCP and PIP joint angle limits, which are 90 and 120 respectively for the index finger.

The Data Glove weights were subsequently calculated as the gradient of the linear least-squares fit representing the relation between the measured joint angles. Although the middle and ring fingers were not measured with Marker Tracking because of visual occlusion, for the purpose of these analyses, the Data Glove measurements of those fingers were compared to the index finger Marker Tracking angles. This is valid, because the middle and ring finger joint angles were approximately equal to those of the index finger, as these fingers were flexed and extended simultaneously and equally during measurements. The pinky and thumb fingers were measured separately.

Following from the analysis, the weights calculated for all fingers are given as follows:

(C2) $$\begin{array}{cccccc} & \text{thumb} & \text{index} & \text{middle} & \text{ring} & \text{pink}\\ \begin{array}{c} w_{DG_{MCP}}\\ w_{DG_{PIP}} \end{array} & [\begin{array}{c} 1.07\\ 1.38 \end{array} & \begin{array}{c} 0.73\\ 2.14 \end{array} & \begin{array}{c} 0.77\\ 2.12 \end{array} & \begin{array}{c} 0.49\\ 1.86 \end{array} & \begin{array}{c} 0.10\\ 1.47 \end{array}] \end{array}$$
